# Physiological Predictors of Operator Performance: The Role of Mental Effort and Its Link to Task Performance

**DOI:** 10.1177/00187208241296830

**Published:** 2024-10-30

**Authors:** Sebastian Pütz, Alexander Mertens, Lewis L. Chuang, Verena Nitsch

**Affiliations:** 1 9165 RWTH Aachen University, Germany; 2 38869 Chemnitz University of Technology, Germany

**Keywords:** supervisory control, adaptive automation, psychophysiology, mental fatigue, mental workload

## Abstract

**Objective:**

The present study investigated how pupil size and heart rate variability (HRV) can contribute to the prediction of operator performance. We illustrate how focusing on mental effort as the conceptual link between physiological measures and task performance can align relevant empirical findings across research domains.

**Background:**

Physiological measures are often treated as indicators of operators’ mental state. Thereby, they could enable a continuous and unobtrusive assessment of operators’ current ability to perform the task.

**Method:**

Fifty participants performed a process monitoring task consisting of ten 9-minute task blocks. Blocks alternated between low and high task demands, and the last two blocks introduced a task reward manipulation. We measured response times as primary performance indicator, pupil size and HRV as physiological measures, and mental fatigue, task engagement, and perceived effort as subjective ratings.

**Results:**

Both increased pupil size and increased HRV significantly predicted better task performance. However, the underlying associations between physiological measures and performance were influenced by task demands and time on task. Pupil size, but not HRV, results were consistent with subjective ratings.

**Conclusion:**

The empirical findings suggest that, by capturing variance in operators’ mental effort, physiological measures, specifically pupil size, can contribute to the prediction of task performance. Their predictive value is limited by confounding effects that alter the amount of effort required to achieve a given level of performance.

**Application:**

The outlined conceptual approach and empirical results can guide study designs and performance prediction models that examine physiological measures as the basis for dynamic operator assistance.

## Introduction

Extensive research has examined the reliability of physiological measures in estimating the mental state of operators engaged in supervisory control. To date, studies have primarily focused on demonstrating that physiological measures are sensitive to changes in task characteristics (Pütz et al., 2024; see also Bafna & Hansen, 2021; Charles & Nixon, 2019; Csathó et al., 2023; Tao et al., 2019) and can discriminate operator states (Ding et al., 2020; Tjolleng et al., 2017; Wilson & Russell, 2003, 2007). Physiological measures could thus provide a continuous and unobtrusive assessment of operators’ mental state, even when adverse changes in operator state have yet to manifest themselves in performance deficits (Sharples & Megaw, 2015). With growing empirical support, researchers have proposed expanding research on physiological measures to explore whether assessing operators’ state could be the basis for predicting operator performance (G. Hancock et al., 2021; Longo et al., 2022; Pütz et al., 2024). This prospect holds appeal from a theoretical and an applied perspective.

**Figure 1.**
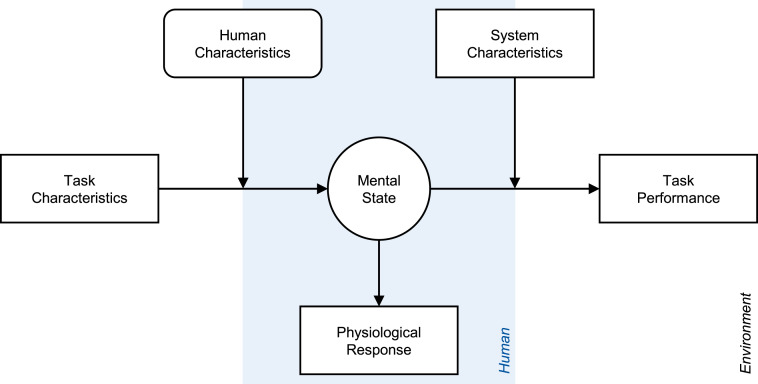
Abstract conceptual model of the role of operators’ mental state. *Note*. The structure of the model is based on the explanatory framework of mental workload by van Acker et al. (2018).

From a theoretical perspective, researchers can treat the operator’s performance as an individual-specific benchmark for physiological measures. Doing so allows them to account for the moderating role of inter-individual differences (i.e., human characteristics) on the relationship between task characteristics and the operator’s mental state (see Figure 1). This moderating influence is neglected when mapping physiological responses directly to changes in task characteristics across individuals, which illustrates the advantage of individual-based compared to group-based analyses (see, e.g., Wilson & Russell, 2007). From an applied perspective, using physiological measures to continuously assess the operator’s ability to perform the task provides the basis for dynamic operator assistance (Aricò et al., 2016; Di Flumeri et al., 2019; Freeman et al., 2004; Prinzel et al., 2003; Wilson & Russell, 2007). This offers a solution to the pitfalls of supervisory control, where task demands can fluctuate from passive monitoring under normal conditions to time-critical decision making in the event of system failures (Endsley, 2017; Sheridan, 2021).

Using physiological measures as predictors of task performance relies on establishing reliable associations between them. However, this can be challenging as different research domains have linked the same physiological responses to different operator states, leading to conflicting implications for their association with performance. This challenge is particularly evident when studies that focus on either *mental fatigue* or *mental workload* are contrasted. Without disentangling these effects, it will be difficult to create predictive models that can be used across research domains. We contribute to the utility of physiological predictors of task performance by demonstrating (1) how using physiological measures as indicators of mental effort—the common factor in mental fatigue and mental workload—can help link these two research domains, and (2) how examining the effect of task characteristics on the association of mental effort and task performance can help align existing empirical findings. Regarding physiological measures, we focus on pupil size and heart rate variability (HRV), the most prominent measures in supervisory control research (Pütz et al., 2024).

### The Role of Mental Effort

With mental effort, we refer to engaging in a task by investing mental resources in service of instrumental behavior (Gendolla & Richter, 2010; Gendolla & Wright, 2009). Thus, we define mental effort in terms of information processing rather than subjective terms (Hockey, 1997; Shenhav et al., 2017). In this sense, mental effort mediates between (1) task characteristics and individual information-processing capacity (i.e., human characteristics) and (2) information-processing fidelity, reflected in task performance (Shenhav et al., 2017). On the one hand, this definition links mental effort to task engagement, which in turn has been defined as the “commitment to effort” (Matthews, 2021, p. 3). On the other hand, it differentiates mental effort from the subjective experience of perceiving a task as effortful. Distinguishing these meanings of the term *effort* (Inzlicht et al., 2018)—*invested effort/task engagement* (positive) and *perceived effort* (negative)—is crucial, as we outline below, the two are often associated in studies on mental workload but dissociate in studies on mental fatigue. The definition is also consistent with the established use of mental effort as correlate of pupil dilation (Kahneman, 1973; van der Wel & van Steenbergen, 2018).

Research on mental fatigue has examined performance declines that result from the prolonged execution of mental tasks. As a key driver of this effect, researchers have identified a decrease in task engagement over time on task (Matthews, 2016, 2021; Matthews et al., 2014, 2017; Reinerman et al., 2006), that is, a decreased commitment to invest mental effort (Hockey, 2011; van der Linden, 2011). This decrease has been attributed to the depletion of mental resources through effort exertion (Baumeister et al., 2007; Warm et al., 2008), a diminishing cost-benefit ratio of performing the task (Boksem & Tops, 2008; Kurzban et al., 2013), and mind-wandering (Smallwood & Schooler, 2006). Notably, the decrease in mental effort invested in the task is often contrasted by an increase in the perceived effort of task execution (Neigel et al., 2020; Warm et al., 2008). On a physiological level, mental fatigue has been associated with decreases in pupil size and task-evoked pupillary responses (e.g., Hopstaken et al., 2015a, 2016; McIntire et al., 2014) and increases in HRV (e.g., Karthikeyan et al., 2022; Matuz et al., 2021; Melo et al., 2017). Thus, research on physiological indicators of mental fatigue has mostly gathered evidence associating impaired task performance with lower mental effort, smaller pupil size, and higher HRV.

Some researchers have examined the role of task engagement and mental effort in mental fatigue by manipulating task reward. They reasoned that increased motivation should counteract the effects of mental fatigue by facilitating task re-engagement and increased mental effort. Indeed, studies have shown that increasing task reward can lead to both retention (Herlambang et al., 2019) and recovery of task performance (Boksem et al., 2006; Hopstaken et al., 2015a, 2015b) as well as reduce the frequency of attentional lapses (Massar et al., 2016, 2019). On a physiological level, increasing task reward has been connected to increases in pupil size and task-evoked pupillary responses (e.g., Herlambang et al., 2019; Hopstaken et al., 2015a, 2015b, 2016), while the evidence on HRV remains limited, lacking conclusive findings (Herlambang et al., 2019). Thus, studies on the effect of task reward on mental fatigue support the aforementioned associations, linking better task performance to higher mental effort and larger pupil size.

Whereas research on mental fatigue often focuses on how mental effort varies over time on task, research on mental workload focuses primarily on how task demands affect operators’ mental effort. The core assumption is that humans cope with higher demands via additional mental effort (Kahneman, 1973; Shenhav et al., 2017), whereby workload refers to the ratio between invested effort and effort capacity (Longo et al., 2022; Young et al., 2015). In this context, there is usually no distinction between invested mental effort and perceived effort, as more demanding tasks require higher levels of information processing and are also perceived as more effortful. Consistent with the mental fatigue literature, the increases in mental effort are typically associated with increased pupil size and decreased HRV (see Charles & Nixon, 2019; Pütz et al., 2024; Tao et al., 2019). However, most studies also find that increasing task demands can impair task performance as the increased demands are not fully compensated by increased mental effort. As a result, the large body of research on physiological indicators of mental workload has mostly found associations of impaired task performance with higher mental effort, larger pupil size, and lower HRV.

**Figure 2.**
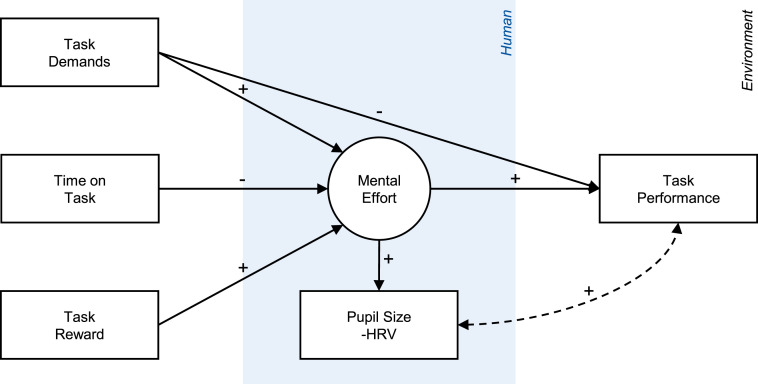
Specified conceptual model of the role of operators’ mental effort. *Note*. Specification of the abstract model in Figure 1 based on the presented synthesis of existing empirical findings across research domains. The model illustrates the expected associations between the variables investigated in the present study. Opposite associations are expected for HRV compared to pupil size.

To summarize, research on mental fatigue and mental workload usually find consistent associations of pupil size and HRV with mental effort but diverging associations of pupil size and HRV with task performance. We propose a synthesis of these findings in Figure 2, which includes the three task characteristics: task demands, time on task, and task reward. All three affect mental effort, which mediates between task characteristics and task performance. Unlike the other two, task demands have a direct effect on task performance by altering the level of mental effort required to achieve a certain level of performance. For example, if an individual invests the same effort despite an increase in task demands, performance will be impaired. Thus, both task demands and mental effort determine performance. Mental effort is associated with physiological responses such as pupil dilation and HRV reduction, which are related to improved task performance due to their common antecedent.

### The Present Study

Given the outlined interdependencies, making reliable predictions of task performance requires information about both task demands and mental effort. Therefore, physiological measures might contribute to performance prediction by (partially) accounting for variance in task performance induced by changes in mental effort. In the present study, we tested this assumption in a process monitoring task. We manipulated task demands and task reward in addition to the progression of time on task to induce variance in participants’ mental effort. We investigated whether the variance in mental effort created covariance in physiological measures and task performance when accounting for the level of task demands. To this end, we first examined whether the three task characteristics showed the expected direct effects on performance and physiological and subjective measures (mental fatigue, task engagement, perceived effort) across participants. This analysis aimed to check the plausibility of mental effort as a viable link between physiological measures and task performance. Second, in our main analysis, we analyzed the intra-individual associations of pupil size and HRV with task performance to estimate their predictive value. Thereby, we tested our research hypothesis *H*: *“Physiological measures, specifically pupil size (a) and HRV (b), can contribute to the prediction of task performance, in the form of response times, when controlling for the level of task demands”*.

## Method

### Participants

Fifty participants (28 women and 22 men; *M*_age_ = 24.34 years, *SD*_age_ = 3.60 years) were recruited at RWTH Aachen University. All participants had (corrected-to-) normal vision, spoke German at a native level, and received 20 € as compensation. This research complied with the American Psychological Association Code of Ethics and was approved by the Ethics Committee at RWTH Aachen University. Informed consent was obtained from each participant.

### Experimental Task

**Figure 3.**
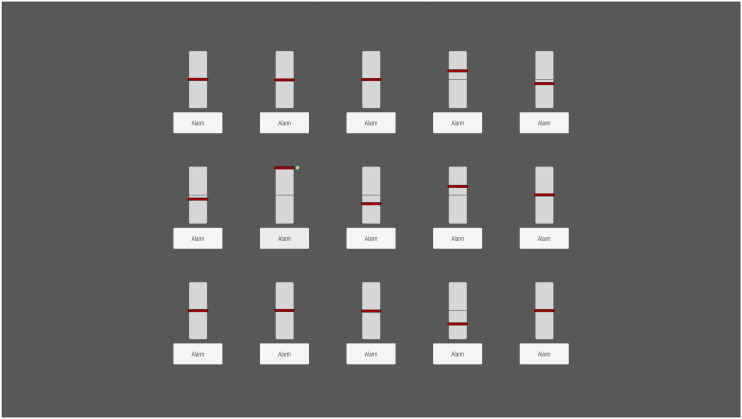
Interface of the process monitoring task. *Note*. The interface consisted of 15 parameter gauges arranged in a three-by-five grid, each with an associated alarm button. In this example, the gauge in row 2 column 2 indicates a critical system event and the small confirmation marker (green circle) next to it indicates that the participant has responded.

A simulated process monitoring task was developed in the Unity game engine (see Figure 3). Participants had to monitor a three-by-five grid of gauges that each indicated the continuous fluctuation of a simulated process parameter around a central value (cf. Shi & Rothrock, 2022; Yang & Kim, 2019). Participants were instructed to detect critical system events, which were defined as one process parameter reaching the lower or upper scale limit, and respond as fast as possible by clicking on the alarm button below the associated gauge. After a correct response, a confirmation marker was presented next to the respective gauge until the end of the event, which lasted 7 s each.

For each participant, a 1 Hz time series of parameter values was sampled for each gauge. Parameter values could remain constant, increase, or decrease between successive timestamps. As the deviation of values from the center increased, the probability of further deviation decreased. Parameter values could not reach the scale limits outside of preselected timestamps. With a preselected event timestamp approaching, the respective parameter value was set to move towards the nearer of the two scale limits. At runtime, values were linearly interpolated between successive timestamps of the sampled time series to display continuous value transitions.

Two task demand levels were implemented. In the low task demand condition, value changes were fixed to one-twelfth of the scale per second, while in the high task demand condition, half of the value changes spanned one-sixth of the scale per second. Therefore, the two task demand levels differed in the consistency and maximum speed of value changes, that is, temporal uncertainty (Szalma & Claypoole, 2019). This made detecting gradual transitions of parameter values towards the scale limits more challenging. The high demand condition also resulted in larger average deviations of parameter values from the center of the scale. These manipulations were established in a pretest to ensure distinct task demand levels and minimize ceiling effects in task performance.

The task demand level alternated between the ten 9-minute task blocks, with half of the participants starting in the low and half in the high task demand condition. The rate of critical system events was set to three events per minute, that is, 27 events per block across all gauges. The timing of events was randomized for each participant and block, with no overlap of events and a minimum offset of 3 s between events. For all participants, the 270 events were evenly distributed among the 15 gauges to minimize systematic differences in gaze positions, which might have affected pupil size estimations. Successive events could not be indicated by the same gauge.

For blocks 9 and 10, a task performance reward was introduced. Participants were told that they would earn points for each response. The maximum number of points was 10, which decreased by 1 point for every 500 ms of response time to a minimum of 1. The earned point value was displayed for 1 s following the response. Participants were instructed that they could earn a bonus of 5 € if they earned more points than the average participant in a fictitious prestudy. In fact, all participants received the bonus at the end of the study. Placing the reward blocks at the end (cf. Hopstaken et al., 2015a; 2015b, 2016) was chosen so that the expected increase in motivation could be separated from mental fatigue effects over time on the task in statistical analyses.

### Apparatus

Participants were seated at a desk in a lit testing room. The desk was flanked by partitions that blocked participants’ view of the rest of the lab space and the experimenter, who remained in the room during the experiment to check data recording. The experimental task was presented on a 27 in. IPS monitor with a resolution of 2,560 × 1,440 pixels at a distance of 70 cm from the participants, with the gauges occupying a 25 × 32 cm area in the center of the screen. The monitor refresh rate was set to 144 Hz, matching the fixed frame rate of the task application. To interact with the application, participants used a standard computer mouse.

Participants’ pupil size was measured by recording their pupil diameters at 60 Hz using an FX3 remote eye tracker running EyeWorks version 3.21 by EyeTracking. Ambient lighting conditions were kept constant across participants. In addition, participants wore a chest strap attached to a Movesense Medical single-channel electrocardiography (ECG) sensor, which has been successfully validated against a conventional 12-channel ECG sensor (Rogers et al., 2022). ECG data was collected at 512 Hz and transmitted via Bluetooth to a smartphone running the Movesense Showcase app version 1.1.

### Measures

#### Performance measures

Response times were used as the primary performance measure. Failure to respond before the end of an event was labeled a miss. The long event duration of 7 s was intended to capture most of the variance in response times. Thus, misses were only considered as a secondary performance measure. To compare effect sizes in the statistical analyses, performance measures were aggregated at the block level by calculating median response times (RT) and miss rates (MR) per participant and block.

#### Physiological measures

Using the Pupil Diameter Analyzer (Kret & Sjak-Shie, 2019) of the PhysioData Toolbox version 0.6.3 (Sjak-Shie, 2022), we preprocessed raw pupil diameter data with the following sequential steps. Lower and upper cut-off values were set to 1.5 mm and 9 mm, respectively. Isolated data clusters were removed if they had durations of less than 50 ms and were separated from other clusters by more than 40 ms. Datapoints with a median absolute deviation (*MAD*) greater than 6 from successive datapoints were removed as dilation-speed outliers. To prevent edge artifacts, we further removed 50 ms of data before and after recorded data gaps of 75 ms–2000 ms. Next, trend-line outliers were identified based on a 16 Hz low-pass filter and an *MAD* threshold of 8, with four iterative filter passes. The remaining data were used to calculate mean pupil diameters across both eyes, interpolated and upsampled to 1000 Hz, and finally smoothed with a 4 Hz low-pass filter. Any gaps larger than 250 ms were not interpolated. This process resulted in a time series of pupil diameter (PD) per block for every participant, which was used to derive the mean values submitted for statistical analysis.

Raw ECG data were preprocessed using Kubios HRV Premium version 3.5 (Tarvainen et al., 2014). Beat detection was followed by noise detection (set to “Medium”), artifact correction (Lipponen & Tarvainen, 2019), and the removal of nonstationary trends in the times series (Tarvainen et al., 2002). The resulting data were used to calculate the square root of the mean squared differences between successive RR intervals (RMSSD) as HRV indicator per participant and block. As a reference, we also report participants’ heart rate (HR) per block as a secondary ECG measure.

#### Subjective measures

Three subjective ratings were collected after each block as references for physiological measures. Mental fatigue (MF) and task engagement (TE) were assessed using single items on a scale from 0 (*not at all*) to 100 (*extremely*; cf. Hopstaken et al., 2016). Perceived effort (PE) was assessed via the Scale of Perceived Effort (Eilers et al., 1986), the German counterpart of the Rating Scale Mental Effort (Zijlstra, 1993). The scale ranges from 0 to 220 with seven scale anchors (from *hardly effortful* to *extraordinary effortful*). Importantly, mental effort, as defined above, is expected to correspond closely with the subjective experience of task engagement rather than perceived effort. Single-item scales were chosen to minimize the disruption of the task flow. All items were presented in German (wordings are available in the Supplementary Data).

### Procedure

Participants were asked not to consume caffeine or nicotine for 4 hours, and alcohol for 12 hours, prior to the study. Upon arrival, participants handed over their smartphones and wristwatches to minimize external distractions. Then, they received written information about the study and provided signed informed consent. They were also informed that they would receive instructions on how to earn a bonus of 5 € later in the experiment. This was followed with preparation for physiological measurements, including eye makeup removal for eye-tracking. Finally, participants received written instructions for the experimental task and performed a 2-minute practice block. The duration of the practice block was examined in the pretest to achieve sufficient stabilization of task performance.

After answering participant questions, the experimenter initiated the experiment and the participants performed the ten task blocks. Participants were instructed to use the gaps between blocks to answer the subjective measure items only, and not to rest. Following block 8, they received short written instructions about the task reward condition. As a result, the average time between blocks 8 and 9 was about 25 s longer (*M* = 53.48 s, *SD* = 30.11 s) than the other between block intervals (*M* = 27.53 s, *SD* = 28.03 s). After finishing all ten blocks, participants completed a postsurvey that included demographic information. Finally, participants were debriefed about the reward procedure and received their full monetary compensation. The entire study took about 2 hours, with the experiment beginning about 20 minutes after the participants entered the room.

### Data Analysis

After screening the performance data for outliers and physiological data for data quality, the main analysis was divided into two steps. First, to establish an overview of the direct effects of the included experimental manipulations, linear mixed models (LMM) were fitted to examine the effect of the three independent variables: task demands (low vs. high), time on task (1–10), and task reward (no reward vs. reward) on the two performance, three physiological, and three subjective measures. All models included interaction terms for task demands with the other two independent variables. Second, to test our hypothesis on the predictive value of physiological measures for task performance, PD and HRV were added sequentially to a baseline LMM of RT while controlling for the level of task demands. The likelihood ratios of the model steps were assessed to determine whether the physiological predictors added significant predictive value.

Model steps were also compared using the Akaike information criterion (AIC) and the Bayesian information criterion (BIC). All LMMs included random intercepts for participants and were fitted with the R (version 4.3.1) package *lme4* (Bates et al., 2015), except for the binomial regression of MR, which was fitted with *glmmTMB* (Brooks et al., 2017). Test statistics were estimated with *lmerTest* (Kuznetsova et al., 2017). For standardized effect sizes, multilevel correlations were computed with *correlation* (Makowski et al., 2020) and conditional $\left(R_{c}^{2}\right)$ and marginal $\left(R_{m}^{2}\right)$ *R*^2^ (Nakagawa et al., 2017) with *performance* (Lüdecke et al., 2021). Whereas $R_{c}^{2}$ takes the variance explained by the random intercept into account, $R_{m}^{2}$ considers only the variance of the fixed effects. The final LMMs for RT and HRV were fitted on log-transformed outcome variables to account for heteroscedasticity in the original model fits.

## Results

### Data Check

We removed blocks from further analysis if they had an MR of 2 *SD*s above the *M*_MR_ of .04 (*SD*_MR_ = .12). In total, we removed 23 blocks, including all data of two participants. Next, the signal coverages of PD time series were assessed. A lower threshold of 70% (see Winn et al., 2018) was set for both the signal coverage per block and the resulting number of valid blocks per participant. This resulted in the exclusion of one participant and five individual blocks for PD analyses. Errors in data transmission caused the loss of ECG data for five participants. In addition, artifacts in the ECG sampling rate causing a bias in the estimated sample duration of more than 1% resulted in the exclusion of data from 17 blocks. Therefore, statistical analyses for (1) performance and subjective ratings were based on data from 477 blocks, (2) PD on 462 blocks, (3) RMSSD (i.e., ECG) on 410 blocks, and (4) the hypothesis test combining PD and RMSSD on 405 blocks.

### Manipulation Check

#### Performance measures

**Figure 4.**
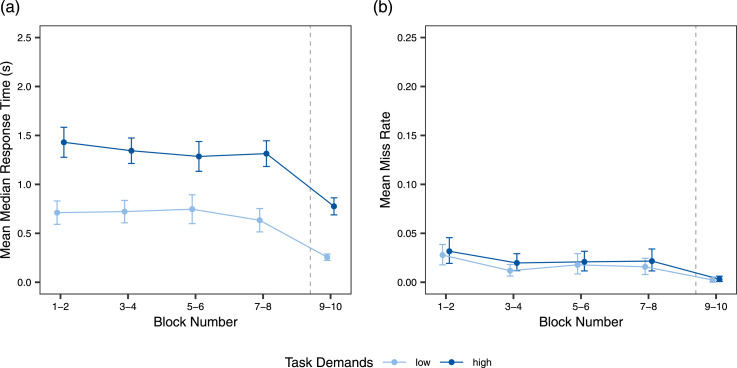
Results for the performance measures. *Note*. Mean median response times (a) and miss rates (b) are displayed as a function of task demands and time on task, as well as task reward in blocks 9 and 10. Blocks are grouped in pairs to account for counterbalancing the order of block demand levels across participants. Error bars indicate 95% confidence intervals (using bootstraping for mean miss rate).

Figure 4 presents mean RT and MR. For RT, there was a significant effect of task demands, with longer RT in the high demand condition, and of task reward, with shorter RT in the reward blocks (see Table 1). The task reward effect was significantly larger in the high task demand condition. The effect of time on task was not significant. For MR, only the main effect of task reward was significant, with fewer misses when task reward was added. All other effects were nonsignificant.

**Table 1.** Linear Mixed Models for Performance Measures.

	*B*	*SE B*	95% *CI*	*p*	$R_{c}^{2}$	$R_{m}^{2}$
Median response time (log(s))					.77	.52
(Intercept)	−0.45	0.07	[−0.59, −0.32]			
Task demands	0.74	0.06	[0.61, 0.86]	<.001		
Time on task	−0.02	0.01	[−0.04, 0.00]	.058		
Task reward	−0.82	0.08	[−0.97, −0.67]	<.001		
Task demands × Time on task	0.01	0.02	[−0.03, 0.04]	.745		
Task demands × Task reward	0.32	0.11	[0.10, 0.53]	.003		
Miss rate					.33	.15
(Intercept)	−4.09	0.24	[−4.56, −3.62]			
Task demands	0.22	0.24	[−0.26, 0.69]	.372		
Time on task	−0.08	0.05	[−0.17, 0.01]	.091		
Task reward	−1.71	0.64	[−2.96, −0.45]	.008		
Task demands × Time on task	0.01	0.06	[−0.12, 0.13]	.905		
Task demands × Task reward	0.00	0.85	[−1.66, 1.66]	.999		

*Note.* Task demands and task reward are dummy coded with *low* and *no reward* as reference, respectively. Time on task is scaled from 0 to 9. Median response time is log-transformed.

#### Physiological measures

**Figure 5.**
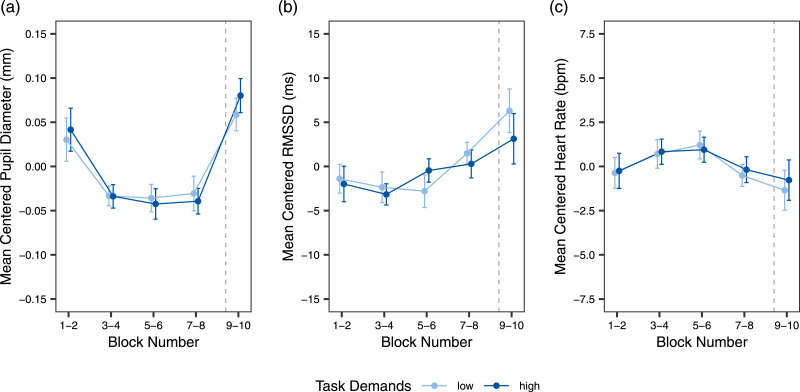
Results for the physiological measures. *Note*. Mean-centered pupil diameters (a), RMSSD (b), and heart rate (c) are displayed as a function of task demands and time on task, as well as task reward in blocks 9 and 10. The variables were centered based on participants’ means to account for the high level of inter-individual differences in physiological indicators. Blocks are grouped in pairs to account for counterbalancing the order of block demand levels across participants. Error bars indicate 95% confidence intervals.

Figure 5 presents mean-centered PD, RMSSD, and HR. PD showed a significant effect of time on task, with a decrease of PD over time on task, and a significant effect of task reward, with larger PD in the reward blocks (see Table 2). The other effects were nonsignificant. For RMSSD, the analysis also yielded significant effects for time on task and task reward. RMSSD increased over time and increased further when task reward was added. The remaining effects yielded nonsignificant results. HR showed only a significant effect of task reward, decreasing as reward was added.

**Table 2.** Linear Mixed Models for Physiological Measures.

	*B*	*SE B*	95% *CI*	*p*	$R_{c}^{2}$	$R_{m}^{2}$
Pupil diameter (mm)					.93	.03
(Intercept)	2.77	0.04	[2.70, 2.84]			
Task demands	0.01	0.01	[−0.02, 0.04]	.443		
Time on task	−0.01	0.00	[−0.01, −0.01]	<.001		
Task reward	0.12	0.02	[0.09, 0.15]	<.001		
Task demands × Time on task	0.00	0.00	[−0.01, 0.00]	.277		
Task demands × Task reward	0.04	0.02	[0.00, 0.08]	.072		
RMSSD (log(ms))					.90	.02
(Intercept)	3.28	0.08	[3.12, 3.43]			
Task demands	−0.02	0.03	[−0.08, 0.05]	.578		
Time on task	0.02	0.01	[0.01, 0.03]	.005		
Task reward	0.11	0.04	[0.03, 0.18]	.009		
Task demands × Time on task	0.01	0.01	[−0.01, 0.02]	.532		
Task demands × Task reward	−0.09	0.06	[−0.20, 0.02]	.107		
Heart rate (bpm)					.94	.00
(Intercept)	81.26	1.82	[77.66, 84.85]			
Task demands	0.06	0.61	[−1.12, 1.25]	.918		
Time on task	0.01	0.10	[−0.19, 0.21]	.918		
Task reward	−1.69	0.73	[−3.12, −0.26]	.021		
Task demands × Time on task	0.00	0.15	[−0.29, 0.28]	.994		
Task demands × Task reward	0.53	1.03	[−1.49, 2.54]	.609		

*Note.* Task demands and task reward are dummy coded with *low* and *no reward* as reference, respectively. Time on task is scaled from 0 to 9. RMSSD is log-transformed.

#### Subjective measures

**Figure 6.**
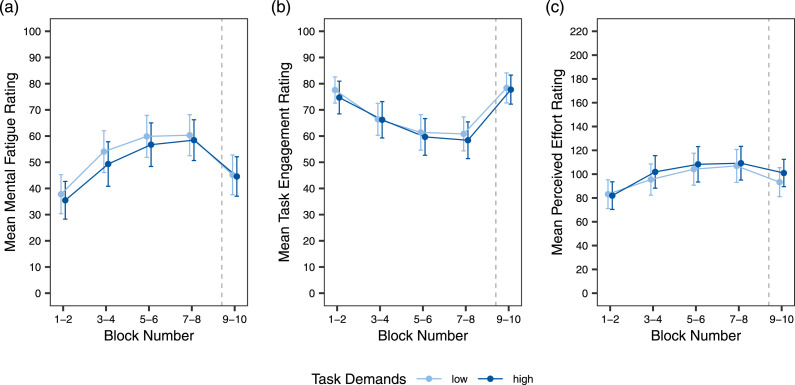
Results for the subjective measures. *Note.* Mean subjective ratings of mental fatigue (a), task engagement (b), and perceived effort (c) are displayed as a function of task demands and time on task, as well as task reward in blocks 9 and 10. Blocks are grouped in pairs to account for counterbalancing the order of block demand levels across participants. Error bars indicate 95% confidence intervals.

Figure 6 presents the mean subjective ratings for MF, TE, and PE. Statistical analyses yielded congruent results across the three variables, with all showing significant effects of time on task and task reward, but no significant effect of task demands or either interaction term (see Table 3). Both MF and PE increased with time on task and decreased when task reward was added. TE showed the opposite effects, decreasing with time on task and increasing in the reward blocks.

**Table 3.** Linear Mixed Models for Subjective Measures.

	*B*	*SE B*	95% *CI*	*p*	$R_{c}^{2}$	$R_{m}^{2}$
Mental fatigue					.67	.09
(Intercept)	40.14	3.79	[32.70, 47.58]			
Task demands	−3.25	3.09	[−9.29, 2.79]	.295		
Time on task	3.70	0.52	[2.68, 4.71]	<.001		
Task reward	−26.45	3.70	[−33.69, −19.22]	<.001		
Task demands × Time on task	0.05	0.74	[−1.40, 1.51]	.945		
Task demands × Task reward	2.34	5.27	[−7.95, 12.62]	.657		
Task engagement					.75	.11
(Intercept)	76.20	3.07	[70.15, 82.24]			
Task demands	−2.06	2.19	[−6.33, 2.22]	.348		
Time on task	−2.82	0.37	[−3.54, −2.10]	<.001		
Task reward	26.17	2.62	[21.05, 31.29]	<.001		
Task demands × Time on task	0.09	0.53	[−0.94, 1.12]	.860		
Task demands × Task reward	0.51	3.73	[−6.78, 7.79]	.892		
Perceived effort					.73	.04
(Intercept)	83.60	6.35	[71.12, 96.08]			
Task demands	1.65	4.49	[−7.11, 10.41]	.713		
Time on task	3.98	0.75	[2.50, 5.45]	<.001		
Task reward	−24.21	5.37	[−34.70, −13.73]	<.001		
Task demands × Time on task	0.37	1.08	[−1.74, 2.48]	.713		
Task demands × Task reward	3.05	7.64	[−11.87, 17.96]	.690		

*Note.* Task demands and task reward are dummy coded with *low* and *no reward* as reference, respectively. Time on task is scaled from 0 to 9.

### Hypothesis Test

Both the addition of PD (*χ*^2^(1) = 21.88, *p* < .001, AIC = 582 compared to 602, BIC = 603 compared to 618) and RMSSD (*χ*^2^(1) = 37.67, *p* < .001, AIC = 547, BIC = 571) significantly improved the model fit for predicting RT (see Table 4), supporting H_a_ and H_b_. The final model yielded significant negative associations of PD and HRV with RT.

**Table 4.** Hypothesis Test: Linear Mixed Model for the Prediction of Median Response Time.

	*B*	*SE B*	95% *CI*	*p*	$R_{c}^{2}$	$R_{m}^{2}$
Step 1					.57	.37
(Intercept)	−0.78	0.06	[−0.89, −0.66]			
Task demands	0.85	0.05	[0.76, 0.94]	<.001		
Step 2					.59	.39
(Intercept)	−0.78	0.06	[−0.89, −0.66]			
Task demands	0.86	0.04	[0.77, 0.94]	<.001		
Pupil diameter	−1.34	0.28	[−1.90, −0.79]	<.001		
Step 3					.63	.43
(Intercept)	−0.78	0.06	[−0.89, −0.66]			
Task demands	0.84	0.04	[0.76, 0.93]	<.001		
Pupil diameter	−1.23	0.27	[−1.76, −0.70]	<.001		
RMSSD	−0.02	0.00	[−0.03, −0.01]	<.001		

*Note.* Task demands are dummy coded with *low* as reference. Pupil diameter and RMSSD are mean-centered per participant to account for inter-individual differences. Median response time is log-transformed.

### Post-Hoc Analysis

To gain a more detailed understanding of the associations between the physiological measures and task performance, we conducted post-hoc analyses to separate the influence of the two task characteristics that affected the physiological measures, that is, time on task and task reward. To this end, we set up two additional LMMs, one for the blocks with no task reward (1–8) and one that compared the two task reward blocks with the two preceding blocks (7–10). For blocks 1–8, the addition of PD did not significantly improve the model fit (*χ*^2^(1) = 3.64, *p* = .057, AIC = 312 compared to 314, BIC = 331 compared to 329), whereas adding RMSSD did (*χ*^2^(1) = 6.30, *p* = .0125, AIC = 308, BIC = 330). The model estimated a nonsignificant positive association between PD and RT, while the significant association between RMSSD and RT remained negative (see Table 5). For blocks 7–10, the model fit was significantly improved by both the addition of PD (*χ*^2^(1) = 20.80, *p* < .001, AIC = 249 compared to 268, BIC = 265 compared to 280) and RMSSD (*χ*^2^(1) = 6.85, *p* = .009, AIC = 244, BIC = 263; see Table 6). The model showed significant negative associations of both PD and RMSSD with RT.

**Table 5.** Post-Hoc: Linear Mixed Model for the Prediction of Median Response Time (Blocks 1–8).

	*B*	*SE B*	95% *CI*	*p*	$R_{c}^{2}$	$R_{m}^{2}$
Step 1					.72	.40
(Intercept)	−0.60	0.06	[−0.72, −0.48]			
Task demands	0.80	0.04	[0.73, 0.87]	<.001		
Step 2					.72	.41
(Intercept)	−0.59	0.06	[−0.71, −0.47]			
Task demands	0.80	0.04	[0.73, 0.87]	<.001		
Pupil diameter	0.51	0.27	[-0.01, 1.04]	.057		
Step 3					.73	.41
(Intercept)	−0.61	0.06	[−0.73, −0.48]			
Task demands	0.80	0.04	[0.73, 0.87]	<.001		
Pupil diameter	0.44	0.27	[−0.08, 0.97]	.097		
RMSSD	−0.01	0.00	[−0.02, 0.00]	.012		

*Note.* Task demands are dummy coded with *low* as reference. Pupil diameter and RMSSD are mean-centered per participant to account for inter-individual differences. Median response time is log-transformed.

**Table 6.** Post-Hoc: Linear Mixed Model for the Prediction of Median Response Time (Blocks 7–10).

	*B*	*SE B*	95% *CI*	*p*	$R_{c}^{2}$	$R_{m}^{2}$
Step 1					.56	.44
(Intercept)	−1.11	0.07	[−1.24, −0.97]			
Task demands	0.98	0.08	[0.82, 1.13]	<.001		
Step 2					.66	.48
(Intercept)	−1.07	0.07	[−1.21, −0.93]			
Task demands	0.99	0.07	[0.85, 1.12]	<.001		
Pupil diameter	−2.20	0.44	[−3.07, −1.30]	<.001		
Step 3					.69	.49
(Intercept)	−1.01	0.07	[−1.16, −0.87]			
Task demands	0.95	0.07	[0.82, 1.09]	<.001		
Pupil diameter	−2.11	0.43	[−2.96, −1.24]	<.001		
RMSSD	−0.01	0.01	[−0.02, 0.00]	.008		

*Note.* Task demands are dummy coded with *low* as reference. Pupil diameter and RMSSD are mean-centered per participant to account for inter-individual differences. Median response time is log-transformed.

### Multilevel Correlations

Table 7 presents multilevel correlation coefficients as standardized estimates of the associations between the primary measures. Correlations are reported for the full dataset (blocks 1–10), when isolating the time on task effect (blocks 1–8), and when focusing on the task reward effect (blocks 7–10). Extending the findings from the post-hoc analysis, the analysis indicated that the associations between the measures observed in the full dataset are mainly attributable to blocks 7–10. Specifically, the directions of the associations observed in blocks 7–10 were consistent with those in the full dataset, whereas the associations in blocks 1–8, that is, when the task reward effect is excluded, partially deviated. For instance, PD had a significant negative association with RT in blocks 7–10 but a positive association in blocks 1–8. PD also exhibited significant correlations with MF and TE. The negative association of RMSSD with RT in the full dataset was mainly present in blocks 7–10. Compared to PD, RMSSD was less correlated with the subjective measures. Subjective measures, particularly MF and TE, correlated with RT.

**Table 7.** Multilevel Correlations Between Measures.

	RT	PD	RMSSD	MF	TE	PE
Blocks 1–10						
Median response time (RT)		−.23***	−.17*	.08	−.25***	.01
Pupil diameter (PD)	−.31***		.03	−.30***	.39***	−.14*
RMSSD	−.37***	.13		−.06	.03	−.06
Mental fatigue (MF)	.25***	−.24***	−.02		−.60***	.58***
Task engagement (TE)	−.37***	.29***	.09	−.55***		−.46***
Perceived effort (PE)	.14*	−.03	.05	.46***	−.33***	
Blocks 1–8						
Median response time (RT)		.20**	−.04	−.06	.03	−.02
Pupil diameter (PD)	.03		−.12	−.44***	.33***	−.37***
RMSSD	−.15	−.04		.10	−.27***	.09
Mental fatigue (MF)	.20**	−.21**	.07		−.65***	.65***
Task engagement (TE)	−.16*	.13	−.10	−.55***		−.60***
Perceived effort (PE)	.13	−.11	.05	.56***	−.48***	
Blocks 7–10						
Median response time (RT)		−.44***	−.10	.42***	−.41***	.24*
Pupil diameter (PD)	−.43***		.08	−.41***	.43***	.04
RMSSD	−.34**	.21		−.19	.24*	−.28*
Mental fatigue (MF)	.43***	−.48***	−.13		−.65***	.48***
Task engagement (TE)	−.55***	.45***	.21	−.65***		−.32**
Perceived effort (PE)	.36***	−.13	.05	.44***	−.30**	

*Note.* Correlations for low task demands blocks are presented in the lower left corner and for high task demands blocks in the upper right corner of the respective table segment. Median response time is log-transformed. *p < .05. **p < .01. ***p < .001.

## Discussion

The data supported our research hypothesis that pupil size and HRV are significant predictors of task performance, that is, response times. Nonetheless, the question remains as to whether they are reliable predictors. Post-hoc analyses revealed nuances of their predictive value, namely, that the associations of physiological measures with performance depended on the data subset. We discuss the implications of these findings by first examining the convergence of the physiological and subjective measures as indicators of participants’ mental effort, followed by illustrating how the task characteristics might have influenced the link between mental effort and task performance.

### Indicators of Mental Effort

The associated trends in pupil size and subjective measures support the established literature, which suggest that pupil size can be an effective index of mental effort (Kahneman, 1973; van der Wel & van Steenbergen, 2018). Blocks with increased pupil size were associated with reports of higher task engagement and accompanied by lower mental fatigue. Specifically, the three measures indicated that spending more time on task decreased the investment of mental effort, and task reward increased the investment of mental effort. Consistent with previous research, the two task characteristics also induced a dissociation between *invested effort/task engagement* and *perceived effort*, as perceived effort showed opposite effects compared to pupil size and task engagement, with ratings increasing over time on task and decreasing with task reward (e.g., Herlambang et al., 2021). Hence, our results support the proposition that increased pupil size indicates higher mental effort in the sense of higher task engagement, not perceiving the task as more effortful. Notably, while all four measures showed the expected effects of time on task and task reward, they were also consistent in showing no task demand effect.

Unlike pupil size, HRV results were inconsistent with subjective measures and prior expectations, as the time on task effect and the task reward effect were in the same direction. Although this pattern of effects is consistent with response times and the inferential analysis indicated a significant association, the observed increase in HRV with task reward casts doubt on the reliability of this finding. HRV is usually expected to decrease rather than increase with higher motivation (Herlambang et al., 2019, 2021). Thus, it seems likely that placing the reward blocks at the end of the experiment confounded the task reward effect with the usual increase in HRV over time on task (Csathó et al., 2023). As the combination of the experimental design and the data prevents distinguishing these effects, the present study provides less clear evidence for HRV compared to pupil size. Accordingly, we base the further discussion of participants’ mental effort on the converging results of pupil size and subjective ratings.

### Effort and Performance

**Figure 7.**
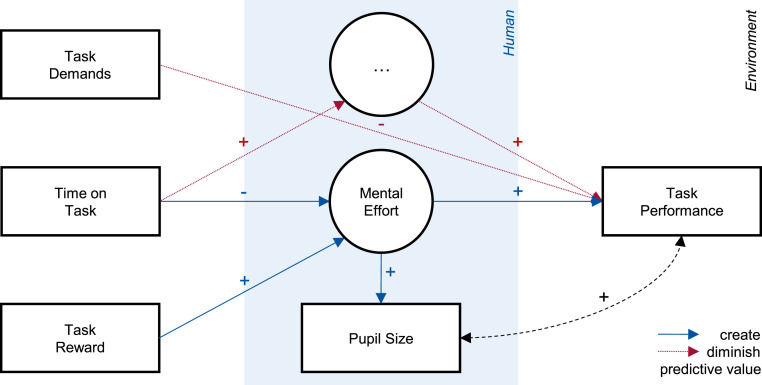
Updated conceptual model of the role of operators’ mental effort. *Note*. Update of the specified model in Figure 2. The model shows the effects found in the present study, illustrating how effects on task performance that are mediated by mental effort create the predictive value of pupil size while effects that are independent of mental effort diminish it. ‘…’ represents latent states that affect task performance but are not reflected in pupil size.

In contrast to the physiological and subjective measures, task performance differed between the two task demand levels, with performance impaired at higher task demands. This suggests that participants did not cope with higher demands via investing more mental effort, that is, higher task engagement, and, thus, could not maintain their performance level. This rationale is plausible in the investigated monitoring task where participants could opt to maintain their effort level as the speed of process parameter variations changed even if they (un)willingly compromised their response latency to critical events. Hence, the analysis supported a direct effect of task demands on task performance but not a mediation through mental effort (see Figure 7). The absence of a task demand effect on mental effort prevented effort and performance from *dissociating* (see P. A. Hancock, 2017; P. A. Hancock & Matthews, 2019) as commonly seen in the mental workload literature, that is, increased effort and pupil size associated with impaired performance. Still, the direct effect of task demands on task performance demonstrated variance in performance to which indicators of mental effort, such as pupil size, are *insensitive*, diminishing their predictive value.

Task performance did not show a significant decrease over time on task, despite the decrease in mental effort indicated by decreasing pupil size and task engagement. Therefore, time on task also induced insensitivity between the variables of interest, with performance being *insensitive* to variations in mental effort. As a result, pupil size was not a significant predictor of task performance when focusing the analysis on the influence of time on task (blocks 1–8). There are multiple conceivable explanations for this observation. For example, the simplicity of the task might have allowed participants to stabilize their performance even when investing less effort, that is, bottom effects in performance. Moreover, the comparatively short practice block might have resulted in learning effects over time on task that reduced the mental effort needed to maintain task performance. Irrespective of the specific explanation, the results show how an effect within the individual that obscures the association between mental effort and performance can diminish the predictive value of pupil size.

Finally, task reward showed the expected effect, as the respective increase in mental effort, indicated by larger pupil size and higher subjective task engagement, was *associated* with increased task performance. Notably, this association created through the task reward manipulation in blocks 9–10 was so strong that it produced the association observed in the full dataset, as can be seen by its absence in data from blocks 1–8. The results demonstrate that, in the absence of confounding effects that alter the amount of effort required to achieve a given level of performance, measures of mental effort, such as pupil size, can be effective predictors of task performance. However, when such confounding effects are present because of changes in the task (e.g., task demands) or the individual (e.g., performance boundaries or task skill), they must be accounted for in statistical modeling, as they otherwise diminish the predictive value of these measures. This takeaway is particularly relevant for research on pupil size and mental workload, where the common practice of manipulating task demands creates precisely such a confounding effect.

Based on the discussed findings, we have derived recommendations for future research on physiological predictors of task performance, which are presented in Table 8.

**Table 8.** Research Recommendations.

No.	Recommendation
1	Conduct dedicated analyses on the association of physiological measures and task performance, mapping intra-individual variance in physiological responses to intra-individual variance in performance.
2	Consider the different meanings of the term effort, that is, task engagement and perceived effort, when comparing physiological measures with subjective ratings.
3	Distinguish between the positive effect of mental effort on task performance and the negative performance effects of variables that tend to increase participants’ mental effort (e.g., high task demands).
4	Account for a wide range of variables that may influence the mental effort invested by participants and the link between mental effort and task performance.

### Limitations

In this article, we have argued for mental effort as a pragmatic solution for linking physiological measures and task performance. We have shown how this approach integrates relevant theory and empirical evidence, and in the discussion section we have illustrated how it can be used to interpret unreliable associations between these variables. However, these interpretations should be treated with caution. Our experiment yielded a complex array of associative, nonassociative, and dissociative patterns between the examined variables, some of which deviated from our a priori assumptions. For example, we did not find reliable associations between pupil size and task performance outside of the task reward blocks. While this observation can be explained within an effort-based account, these explanations rely on post-hoc rationalizations (see differences between Figures 7 and 2) that require further empirical investigation and validation.

In addition, there are explanatory approaches in the literature for associations of physiological measures with task performance other than mental effort. For example, physiological measures have been used as indicators of a general arousal state that correlates with operators’ stress. Following this approach, the association between pupil size and task performance in the task reward blocks could be interpreted as participants being in a more performance-conducive arousal state. Here, the present study cannot provide definitive evidence for or against the potential, partially overlapping conceptual accounts. In fact, making such distinctions is hampered by the need to adhere to observational analyses when examining associations between physiological measures and performance, as neither can be directly manipulated as part of an experimental design. Furthermore, the theoretical nature of the psychological constructs and the indirect relationship of the physiological responses with cognitive states and processes make establishing one-to-one relations difficult or even conceptually unlikely. Thus, only the accumulation of evidence in future empirical research can conclusively answer the question of which conceptual account is most beneficial for describing and predicting associations between physiological measures and task performance in the search for physiological performance predictors.

Regarding our statistical analyses, we used a comparatively long time interval to aggregate physiological data. We did so to compare time intervals with the same number of critical events that we could match to per-block subjective ratings and to conduct joint analyses for pupil size and HRV, as the latter requires longer measurement intervals for reliable estimates. This approach allowed us to investigate overarching effects in associations between physiological measures and task performance to examine their value in assessing operators’ current ability to perform the task. That said, optimizing the length of the time interval provides further opportunities to get more precise estimates of physiological measures’ predictive potential. Moreover, we opted for linear relationships in statistical modeling as they were most suitable for the obtained data. However, researchers should also consider the possibility of nonlinear associations (e.g., van den Brink et al., 2016), especially in the investigation of operator overload.

## Conclusion

Based on previous empirical findings, we have outlined how mental effort can serve as the necessary conceptual link between physiological measures and task performance, allowing for consistent interpretations across different domains of human factors research. On this basis, the present study indicated that physiological measures, specifically pupil size, can make a meaningful contribution to the prediction of task performance by capturing performance changes induced by variations in mental effort. However, the empirical findings also highlight the need to account for confounding effects that alter the association between effort and performance. This will be necessary to make reliable progress in establishing physiological performance predictors and using them for dynamic operator assistance.

## Key Points

• Pupil size effectively captured changes in mental effort, indicating decreases over time on task and increases with the addition of task reward.
• HRV results were inconclusive, as the effects of time on task and task reward were confounded and did not match subjective ratings.
• Both pupil size and HRV significantly contributed to the prediction of task performance.
• Task demands and time on task introduced confounding effects on the link between mental effort and task performance.

## Supplemental Material

Supplemental Material - Physiological Predictors of Operator Performance: The Role of Mental Effort and its Link to Task Performance Supplemental Material for Physiological Predictors of Operator Performance: The Role of Mental Effort and its Link to Task Performance by Sebastian Pütz, Alexander Mertens, Lewis L. Chuang, and Verena Nitsch in Human Factors.
